# Stratification and Storage of Soil Organic Carbon and Nitrogen as Affected by Tillage Practices in the North China Plain

**DOI:** 10.1371/journal.pone.0128873

**Published:** 2015-06-15

**Authors:** Xin Zhao, Jian-Fu Xue, Xiang-Qian Zhang, Fan-Lei Kong, Fu Chen, Rattan Lal, Hai-Lin Zhang

**Affiliations:** 1 College of Agronomy & Biotechnology, China Agricultural University; Key Laboratory of Farming System, Ministry of Agriculture of China, Beijing, China; 2 College of Agronomy, Sichuan Agricultural University, Chengdu, China; 3 Carbon Management & Sequestration Center, School of Environment and Natural Resources, The Ohio State University, Columbus, Ohio, United States of America; Chinese Academy of Sciences, CHINA

## Abstract

Tillage practices can redistribute the soil profiles, and thus affects soil organic carbon (SOC), and its storage. The stratification ratio (SR) can be an indicator of soil quality. This study was conducted to determine tillage effects on the profile distribution of certain soil properties in winter wheat (*Triticum aestivum* L.) and summer maize (*Zea mays* L.) systems in the North China Plain (NCP). Three tillage treatments, including no till (NT), rotary tillage (RT), and plow tillage (PT), were established in 2001 in Luancheng County, Hebei Province. The concentration, storage, and SR of SOC and soil total nitrogen (TN) were assessed in both the wheat and maize seasons. Compared with RT and PT, the mean SRs for all depth ratios of SOC under NT increased by 7.85% and 30.61% during the maize season, and by 14.67% and 30.91% during the wheat season, respectively. The SR of TN for 0–5:30–50 cm increased by 140%, 161%, and 161% in the maize season, and 266%, 154%, and 122% in the wheat season compared to the SR for 0–5:5–10 cm under NT, RT and PT, respectively. The data indicated that SOC and TN were both concentrated in the surface-soil layers (0–10 cm) under NT but were distributed relatively evenly through the soil profile under PT. Meanwhile, the storage of SOC and TN was higher under NT for the surface soil (0–10 cm) but was higher under PT for the deeper soil (30–50 cm). Furthermore, the storage of SOC and TN was significantly related to SR of SOC and TN along the whole soil profile (*P*<0.0001). Therefore, SR could be used to explain and indicate the changes in the storage of SOC and TN. Further, NT stratifies SOC and TN, enhances the topsoil SOC storage, and helps to improve SOC sequestration and soil quality.

## Introduction

Soil quality is the capacity to realize the functions of a specific type of soil, within natural or managed ecosystem boundaries, to sustain plant and animal productivity, maintain or enhance water and air quality, and support human health and habitation [1]. Farm-management strategies can deteriorate, stabilize or improve soil-ecosystem functions and thus influence soil quality [2]. Soil organic matter (SOM) is a key indicator that impacts soil quality as well as the crop growth and production [2–4]. The concentration of SOM in the soil surface plays vital roles in erosion control, the conservation of nutrients, water infiltration and other important soil functions [2]. Additionally, soil organic carbon (SOC) sequestration, which also impacts soil quality, is an important strategy to mitigate climate change and improve crop production [5–7]. Furthermore, stratification of SOM in surface soil can function as a vital interface to control the exchange of substance into and out of the soil which is essential to maintain soil quality [2, 8, 9]. Thus, soil surface SOM and SOC sequestration is crucial to soil quality and agricultural productivity.

Most previous studies have indicated that conservation tillage (CT, e.g., no-till, NT) can increase the concentrations of SOC and soil total nitrogen (TN) in the surface layer compared with conventional tillage (e.g., plow tillage, PT) [8, 10–13]. This enrichment of the surface layer with SOM maintains soil quality by enhancing soil aggregation and improving aeration [8]. The magnitude of SOC sequestration is affected by the tillage practices and the associated changes in the soil-surface environment [5, 7]. However, it is argued that increasing in SOC under NT did not result from a net increase in SOC storage and sequestration capacity but the redistribution of C near to the soil surface [14–17]. Indeed, the storage of SOC and TN were increased at surface soil but decreased at deep soil under NT compared to conventional tillage [15, 18]. As for the entire soil profile (e.g., 0–50 cm), the storage of SOC and TN were observed that no significance or even lower under NT than that under conventional tillage [13, 19]. Therefore, the controversial results of tillage-induced changes in SOC concentration and storage may lead to a misunderstanding of tillage practices effects on soil function. In addition, variation in different soil types, climatic conditions, and cropping systems will also add the difficulty to reach a coherence conclusion that how tillage practices affect soil quality.

The stratification ratio (SR) is a ratio of the parameters (e.g., SOC, total nitrogen, soil bulk density, and etc.) in the soil surface to those in a deeper layer [2]. Generally, deeper depths are used to normalize the assessment and make valid comparisons among soils from different eco-regions or landscape positions with inherent differences in the soil capability [3], e.g., the bottom of the plow layer, and to describe the distribution and stratification of the soil properties. Franzluebbers [2] concluded that an SR>2 would be uncommon under degraded conditions. Due to the differences in the soil-nutrient distribution by various management strategies, stratification of SOM (e.g., SOC and TN) has been reported as an important indicator of soil quality [2, 3, 20]. The index of SR allows kinds of soils to be compared on the same assessment scale because of an internal normalization procedure that accounts for inherent soil differences [2]. Furthermore, SR also emphasizes a surface beneficiation of SOM, which is vital to receive input nutrient, catch rainfall, partition flux of gases into and out of soil, more to soil quality and SOC sequestration capacity than just a high total standing storage of SOM [2–4, 13, 21]. A high SR of SOC could be an efficient indicator of the dynamic soil quality and SOC sequestration, regardless of the soil type and climatic regime [2, 3]. Previous studies have shown that the SR varied from 1.0 to 1.9 under conventional tillage and from 1.5 to 4.1 under NT [2, 3, 8, 22, 23], but no specific or consistent value of SR has been observed to indicate a high soil quality. As concluded by Franzluebbers [2], further research is need to enhance the usage of SR of SOM as a soil quality indicator.

Tillage practices can affect the SR and ultimately soil quality due to a different distribution pattern of the soil properties. The adoption of CT can alter the soil properties by soil depth, e.g., SOC [24, 25], TN [11, 26], the enzyme activities [27], and the soil C:N ratio [22]. In contrast, plow tillage (PT) can cause a uniform distribution of SOM [2, 22]. Additionally, conversion from conventional tillage to NT can result in a redistribution in the SOC with soil depth and enhance the SOC sequestration [10, 28], particularly in the surface-soil layer [8, 10]. Therefore, soil quality is maintained under NT system by enhancing aggregation and facilitating aeration, mainly because of the enrichment of the surface SOC [8].

The North China Plain (NCP), with a predominant double cropping system of winter wheat (*Triticum aestivum* L.) and summer maize (*Zea mays* L.), is one of the most important agricultural regions in China. However, several constraints exist that limit the agricultural development (e.g., water-resource scarcity, labor shortage, and low economic benefit) [18, 29]. Recently, the CT system as a C-smart practice has been adopted and popularized instead of the traditional system (e.g., PT) in this region due to its ability to enhance the SOC sequestration and its other benefits to the environment and crop production [28]. Du et al. [19] reported that NT enhanced the SR of SOC and TN in this region compared to that of PT; however, it did not increase the SOC and total N storage in the soil profile (0–50 cm). For some other previous studies conducted in this area also illustrated that CT could increase the surface SOC and TN content [30], enhance the net SOC sequestration rate [31], and improve the soil liable C pools [32]. Consequently, an assessment of the SR of SOM under various tillage practices is important to identify strategies for the sustainable management of soil resources in the NCP. Therefore, the objectives of this study were to investigate the stratification of SOC and TN, compare the differences in the SR of these soil properties for the wheat and maize seasons under various tillage systems, and assess the applicability of utilizing SR as an indicator of SOC sequestration and soil quality in the NCP.

## Materials and Methods

### Ethics Statement

This research was performed in cooperation with China Agricultural University and Luancheng Agro-Ecosystem Experimental Station. The farm operations of this experiment were similar to rural farmers’ operations and did not involve endangered or protected species. The experiment was approved by the Key Laboratory of Farming System, China Agricultural University and Luancheng Agro-Ecosystem Experimental Station.

### Experimental site description

The experiment was initiated in 2001 at the Luancheng Agro-Ecosystem Experimental Station (37°50′N, 114°40′E, elevation of this site is 50.1 m) of the Chinese Academy of Sciences, Hebei Province, in the NCP. The Experimental Station is located in piedmont of the Taihang Mountains. The annual average precipitation in this area is approximately 480.7 mm, 70% of which occurs during the summer, and the mean annual air temperature is 12.2°C. The predominant soil type is a silt loam with 13.8% sand, 66.3% silt, and 19.9% clay, 1.4 g cm^-3^ bulk density (ρ_b_) in the plow layer and 9.11 g kg^-1^ of SOC in the top soil (0–10 cm). The principal soil properties for the 0–30 cm depth are listed in Table 1. The dominant cropping system in this region consists of winter wheat and summer maize. The growing season of the winter wheat is from mid-October to early June, whereas that of the summer maize is from mid-June to early October. Before the experiment, the tillage systems were moldboard plow tillage (PT) for winter wheat and no-till (NT) for summer maize in 1990s.

**Table 1:** Principle soil properties before treatment.

Soil depth (cm)	ρ_b_ (g cm^-3^)	TN (g kg^-1^)	AN (mg kg^-1^)	K (mg kg^-1^)	P (mg kg^-1^)	SOM (mg kg^-1^)
0–10	1.34	0.74	37.95	115	62.90	12.4
10–20	1.42	0.64	30.58	90	39.62	12.8
20–30	1.64	0.45	27.99	65	23.32	9.9

ρ_b_, soil bulk density; TN, total nitrogen; AN, alkali-hydrolyzable nitrogen; K, available potassium; P, phosphorus; SOM, soil organic matter.

### Experimental design

The three tillage treatments included PT, RT and NT. Each tillage treatment was replicated three times, and the area of each plot was 560 m^2^ (8 x 70 m). The various tillage practices were implemented only in the winter wheat season, whereas direct seeding without any tillage was performed in the summer maize season for all of the treatments. The maize residues were chopped twice with a residue pulverizer for all treatments. The winter wheat was harvested by a combine harvester, and approximately 30 cm height of the wheat residue was retained in the fields in all of the treatments. The PT treatment was plowed once to a depth of 20 cm with a moldboard plow and then rotavated once to a depth of 8–10 cm before seeding. The RT treatment was rotavated twice to a depth of 8–10 cm. The residues were retained in the fields for all treatments as an average quantity of 6637, 6540, and 5966 kg ha^-1^ yr^-1^ for wheat and 9191, 9078, and 8573 kg ha^-1^ yr^-1^ for maize under PT, RT, and NT, respectively. The NT treatment involved seeding with a NT planter, which cut the residue, opened a small slot for seed placement, and applied fertilizer. The seeding rate was 150 kg ha^-1^ for the wheat and 50 kg ha^-1^ for the maize. Fertilizers for the winter wheat were applied at rates of 130 kg N ha^-1^ and 121 kg P ha^-1^ during sowing and another 138 kg N ha^-1^ at the regreening stage. Shortly after jointing, the summer maize was topdressed at the rate of 210 kg N ha^-1^. The winter wheat was irrigated with the pumping of groundwater for three times (at sowing, the green-turning stage and the jointing stage), at approximately 40–50 mm per irrigation. The summer maize was irrigated only when the rainfall was limited, and the amount of irrigation was the same as that for the winter wheat. To effectively control weeds and insects, herbicides and insecticides were applied one extra time under NT than under CT and RT during the winter wheat season.

### Soil sampling and analysis

Soil samples for SOC and TN were collected in triplicates in October (maize harvest time) of 2009 and June (wheat harvest time) of 2010. Soil bulk density was determined by the core method using a stainless steel ring (5 cm high and 5 cm in diameter) for 0–5, 5–10, 10–20, 20–30, and 30–50 cm depths. Soil samples were oven dried at 105°C for 24 hr to obtain the dry weight [33]. The soil samples were obtained from the 0–5, 5–10, 10–20, 20–30, and 30–50 cm depths, and a composite sample from all replicates was obtained for each depth. The soil samples were air-dried, gently ground, and sieved (2 mm) in the laboratory. The concentration of SOC (g kg^-1^) was determined using the potassium dichromate oxidation titration method [34], and that of TN (g kg^-1^) was determined by the Kjeldahl method [35].

### Stratification ratio calculation

The SRs of SOC and TN were calculated by dividing the concentration determined for each soil property in the 0–5 cm layer by those in the 5–10, 10–20, 20–30 and 30–50 cm layers, following the procedure of [2].

### SOC and TN storage calculation

The SOC and TN storage was calculated by the equivalent soil mass method. The equivalent soil mass SOC and TN storage were computed following Eq (1):

$$M_{element}\;=\;\left[\overset{n}{\underset{i\;=\;1}{\sum }}M_{soil,\;i}\times conc_{i}+\left(M_{j}-\sum_{i\;=\;1}^{n}M_{soil,i}\right)\times conc_{extra}\right]\times 0.001$$ (1)

when *i* = 1, 2, 3, 4, and 5, this represents the 0–5, >5–10, >10–20, >20–30, and >30–50 cm soil depths, respectively. *M*_*element*_ (Mg ha^-1^) is the equivalent soil mass of the SOC and TN storage. *conc*_*i*_ is the concentration of SOC and TN in the soil depth. *conc*_*extra*_ is the extra SOC and TN concentration, and when *i* = 5, the *conc*_*extra*_ was assumed to be equal to the soil depth of 30–50 cm because SOC and TN changed little in the deeper soil.*M*_*j*_ is the certain soil mass, and when *j* = 1, 2, 3, 4, and 5, it represents the maximum soil mass under the various tillage treatments in the 0–5, 0–10, 0–20, 0–30, 0–50 cm soil depths. *M*_*soil*,*i*_ (Mg ha^-1^) was the soil mass of the soil depth, calculated according to Eq (2):

$${\;M}_{soil,\;i}\;=\;\rho_{b,i}\times T_{i}\times 10000$$ (2)

where *ρ*_*b*_ (Mg m^-3^) is the soil bulk density, and *T*_*i*_ (m) is the thickness of the soil depth.

### Data analysis

The SPSS 16.0 analytical software package (SPSS Inc., Chicago, IL, US) was used for comparing the ANOVA. The concentration of SOC, TN and the SOC and TN storage were analyzed with ANOVA for the same depth under various tillage treatments and for the same tillage treatment under various soil depths. Differences among the treatments and soil depths were considered to be significant using the LSD test at *P*<0.05.

## Results

### Soil bulk density

**Fig 1:**
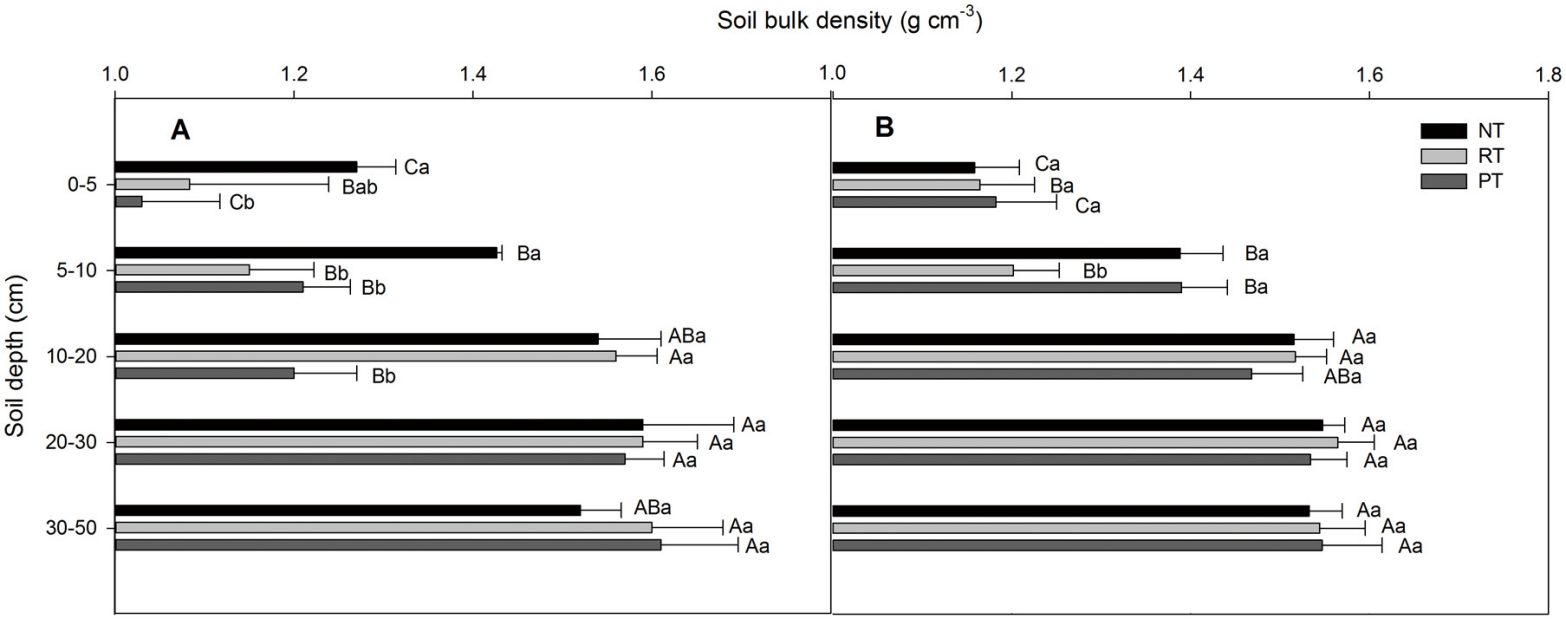
Depth distribution of the soil bulk density (ρ_b_) under different tillage practices. No till (NT), rotary tillage (RT) and plow tillage (PT); (A) after the maize harvest and (B) after the wheat harvest. The data are presented as the means ± SD (n = 3). The various capital letters indicate significant differences among the soil depths, and the lowercase letters indicate significant differences among the treatments (*P*<0.05).

The ρ_b_ significantly increased with increasing soil depth of top 30 cm in both the maize and wheat seasons under NT, RT, and PT (*P*<0.05, Fig 1). However, no statistically significant difference was observed for depths of 20–30 and 30–50 cm. During the maize season, the ρ_b_ ranged from 1.27 (0–5 cm depth) to 1.52 (30–50 cm depth) g cm^-3^ under NT, 1.08 to 1.55 g cm^-3^ under RT, and 1.03 to 1.61 g cm^-3^ under PT (Fig 1A). However, in the wheat season, the ρ_b_ ranged from 1.16 to 1.53, 1.16 to 1.53, and 1.18 to 1.55 g cm^-3^ under NT, RT, and PT, respectively (Fig 1B). Among the tillage systems, NT significantly increased the ρ_b_ at the 0–5 cm depth by 17.23% and 23.30% higher than that under RT and PT at the 0–5 cm depth in the maize season (*P*<0.05), respectively. Additionally, the ρ_b_ was significantly increased by 24.06% and 17.91% under NT and RT and PT at the 5–10 cm depth in the maize season. Furthermore, the ρ_b_ was significantly decreased under PT relative to that under NT and RT in the maize season. Yet, for the deeper soil, no statistically significant difference was observed among the three treatments at depths of 20–30 and 30–50 cm in the maize season. No significant difference in ρ_b_ was observed among NT, RT, and PT for the 0–5, 10–20, 20–30, 30–50 cm depths in the wheat season. Although, ρ_b_ decreased significantly under RT compared with NT and PT at the 5–10 cm depth in the wheat season.

### Concentrations of SOC and TN

**Fig 2:**
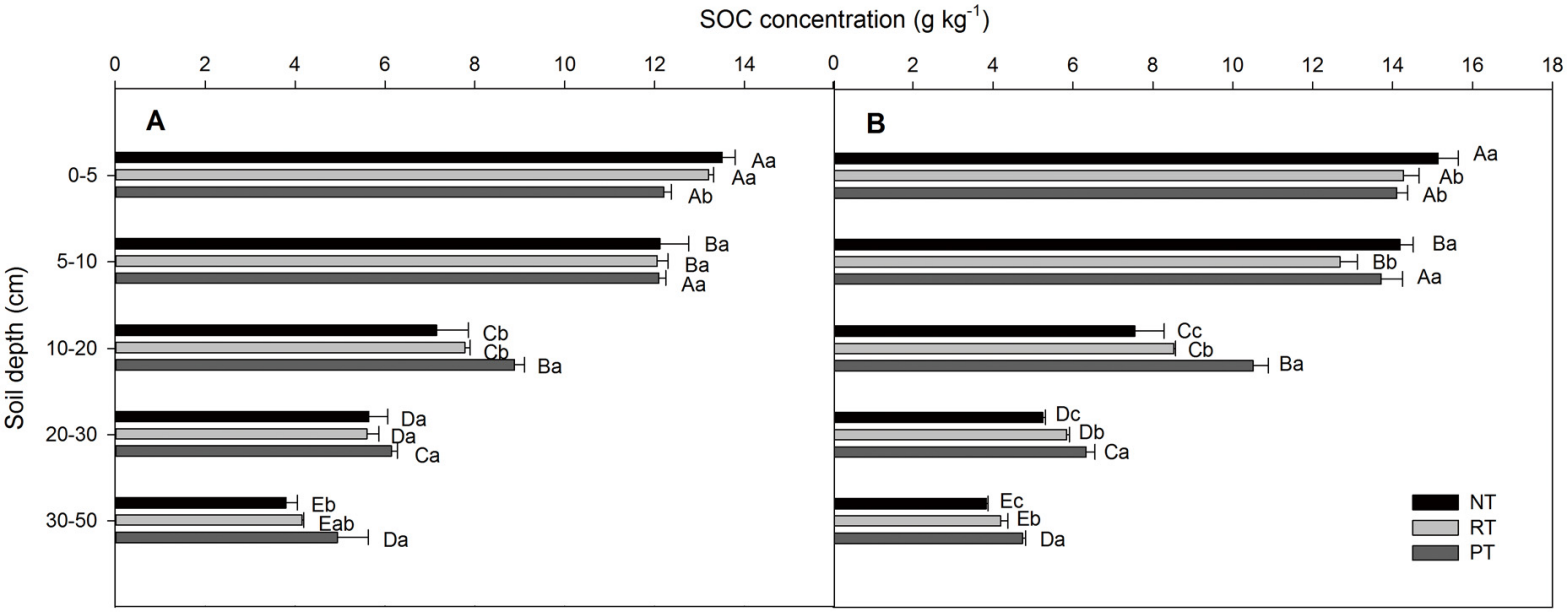
Depth distribution of the soil organic carbon (SOC) concentration under different tillage practices. No till (NT), rotary tillage (RT), and plow tillage (PT): (A) after the maize harvest and (B) after the wheat harvest. The data are presented as the means ± SD (n = 3). The various capital letters indicate significant differences among the soil depths, and the lowercase letters indicate significant differences among the treatments (*P*<0.05).

The concentrations of SOC decreased with increasing soil depth, and the depth distribution of SOC differed among treatments in both the wheat and maize seasons (Fig 2). At the wheat harvest, the SOC concentrations were the highest under NT at the 0–5 and 5–10 cm depths, but the highest under PT at the 10–20, 20–30, and 30–50 cm depths. Furthermore, the SOC concentrations under RT fell between those for PT and NT at all soil depths except 5–10 cm. At the maize harvest, a similar trend as for the wheat harvest was observed among the treatments. Comparing to the maize harvest, the SOC concentrations under NT increased by 12.17%, 17.13%, and 5.56% at the 0–5, 5–10 and 10–20 cm depths, respectively, but decreased by 6.97% at the 20–30 cm depth at the wheat harvest. Similar trends were also observed under RT and PT, for which SOC increased at the 0–5, 5–10 and 10–20 cm depths and decreased or remained constant for the 20–30 and 30–50 cm depths.

**Fig 3:**
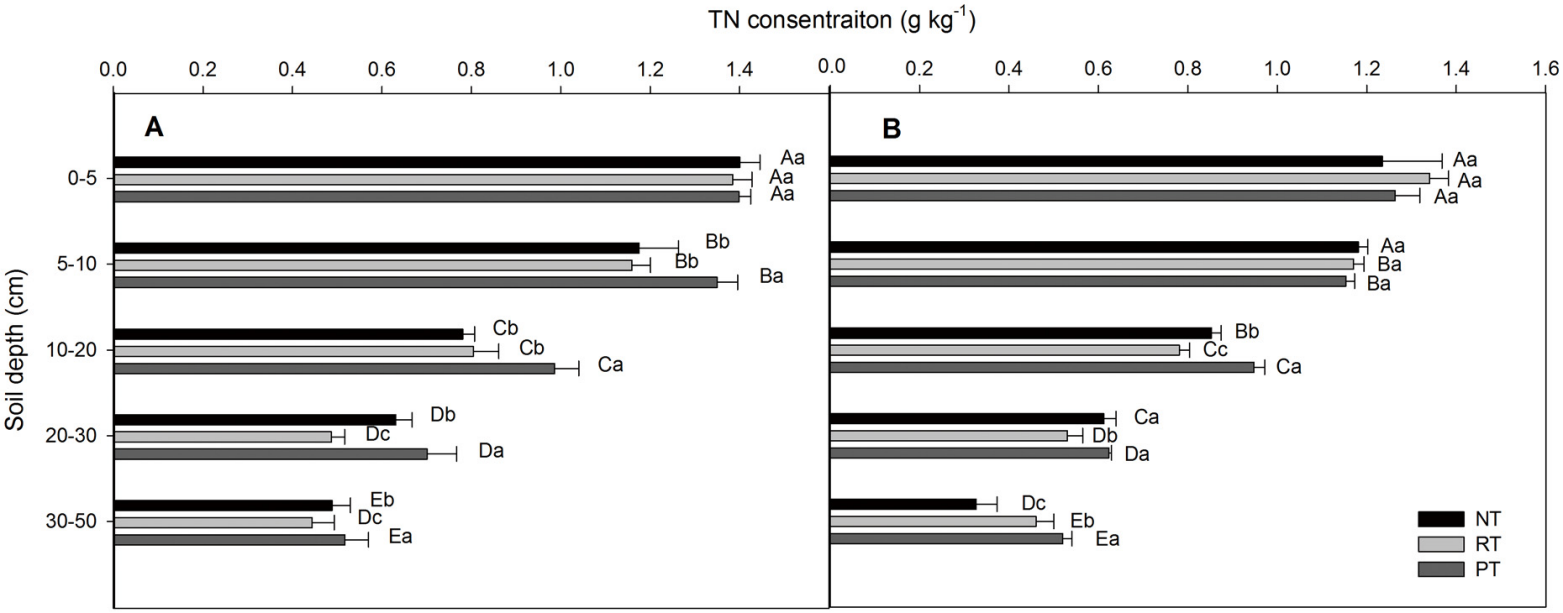
Depth distribution of the soil total nitrogen (TN) concentration under different tillage practices. No till (NT), rotary tillage (RT), and plow tillage (PT): (A) after the maize harvest and (B) after the wheat harvest. The data are presented as the means ± SD (n = 3). The various capital letters indicate significant differences among the soil depths, and the lowercase letters indicate significant differences among the treatments (*P*<0.05).

The soil total nitrogen (TN) concentrations decreased with increasing soil depth under all treatments, both at the wheat and maize harvests (Fig 3). In general, no significant difference was observed in the 0–10 cm layer among the treatments. However, the TN concentration was the highest in the 10–20, 20–30, and 30–50 cm layer under PT. At the wheat harvest, the TN concentration ranged from 1.23 to 0.33 g kg^-1^ under NT, 1.34 to 0.46 g kg^-1^ under RT and 1.26 to 0.52 g kg^-1^ under PT. The TN concentration increased by 8.58% and 6.11% under RT than that under NT and PT at the 0–5 cm depth, respectively. Furthermore, the TN concentration was the highest under NT, 0.90% and 2.44% higher than that under RT and PT at the 5–10 cm depth, respectively, however with no significant. The concentration of TN in PT increased by 11.14%, 1.75%, and 59.04% compared to NT and by 21.43%, 17.47%, and 12.93% compared to RT for the 10–20, 20–30, and 30–50 cm depths, respectively. At the maize harvest, the TN concentrations were the highest at the 5–10, 10–20, 20–30 and 30–50 cm depths under PT. Compared with the NT, the TN concentrations under RT decreased by 1.15%, 1.35%, 29.51%, and 9.95% at the 0–5, 5–10, 20–30, and 30–50 cm depths, respectively, but increased by 2.89% at the 10–20 cm depth.

### Storage of SOC and TN

The SOC storage under NT was significantly higher than that under PT in the surface soil (top 10 cm) by 10.82% and 8.16% at the 0–5 and 0–10 cm depths, respectively (*P*<0.05, Table 2). However, no statistically significant differences were observed among the three treatments in the 0–50 cm soil profile in the maize season, and the SOC storage in this soil layer was 3.82% and 6.28% higher than that under NT and RT, respectively. Additionally, the SOC storage was greater under RT than that under PT in the 0–5 cm layer by 6.53%, but no statistically significant differences existed between the two treatments in the 0–10, 0–20, 0–30, and 0–50 cm layers. In the wheat season, the SOC storage was still higher under NT than that under PT in the 0–5 cm (*P*<0.05) and 0–10 cm (no statistical significance) layers by 7.19% and 4.26%, respectively. Furthermore, the SOC storage increased more under PT when the soil depth increased from the 0–5 to 0–50 depth than that under NT or RT. For the 0–50 depth, the SOC storage was significantly greater under PT by 5.54% than that under NT and by 6.98% than that under RT. Moreover, the SOC storage was significantly higher under NT than that under RT at 0–5 and 0–10 cm, however, no significant differences existed at the 0–20, 30, and 50 depths.

**Table 2:** Storage of the soil organic carbon (SOC) and total nitrogen (TN) for the wheat and maize seasons.

		Maize season			Wheat season		
	Soil depth (cm)	NT	RT	PT	NT	RT	PT
SOC storage (Mg ha^-1^)	0–5	8.57Ea	8.28Eb	7.73Ec	8.94Ea	8.42Eb	8.34Eb
	0–10	25.85Da	23.89Db	23.90Db	28.56Da	25.23Db	27.40Da
	0–20	36.87Ca	35.11Cb	35.72Cab	39.91Cb	37.55Cc	43.05Ca
	0–30	45.83Ba	43.51Bb	44.65Bab	48.03Bb	46.32Bb	50.63Ba
	0–50	57.39Aa	56.14Aa	59.67Aa	59.79Ab	59.17Ab	63.30Aa
TN storage (Mg ha^-1^)	0–5	0.89Ea	0.86Ea	0.88Ea	0.73Ea	0.79Ea	0.75Ea
	0–10	2.57Da	2.38Db	2.69Da	2.37Da	2.34Da	2.35Da
	0–20	3.77Cb	3.51Cc	4.01Ca	3.65Ca	3.47Cb	3.76Ca
	0–30	4.77Bb	4.27Bc	5.00Ba	4.60Ba	4.28Bb	4.72Ba
	0–50	6.26Ab	5.62Ac	6.57Aa	5.60Ab	5.69Aab	5.92Aa

NT, no till; RT, rotary tillage; PT, plow tillage. The data are presented as the means (n = 3). The various capital letters indicate significant differences among the soil depths, and the lowercase letters indicate significant differences among the treatments (P<0.05).

In the maize season, the TN storage increased by 6.03 times under NT, by 5.53 times under RT, and by 6.47 times under PT at the 0–50 cm depth compared to the corresponding values at the 0–5 cm depth. For the surface soil (the 0–5 and 0–10 cm soil depths), no statistically significant differences existed among the three treatments. However, the TN storage followed the order of PT>NT>RT at 0–20, 30, and 50 cm with the significance (*P*<0.05). At the 0–50 cm soil profile, the TN storage under PT was 5.01% higher than that under NT and 16.93% higher than that under RT. Furthermore, the TN storage also exhibited no statistically significant differences among the three treatments at the 0–5 and 10 depths at the wheat harvest. However, for the 0–50 cm soil profile, the TN storage was5.68% higher under PT than that under NT and 3.98% higher under PT than that under RT.

### Stratification of SOC and TN

The SR of SOC increased significantly with increased soil depth, and they differed significantly among both the seasons and treatments (*P*<0.05, Table 3). At the wheat harvest, the SR of SOC ranged from 1.07 to 3.95, 1.13 to 3.41, and 1.03 to 2.98 for NT, RT, and PT, respectively for 0–5:5–10, 0–5:10–20, 0–5:20–30, and 0–5:30–50 cm. The SR of SOC for 0–5:5–10 cm under RT was significantly higher than that under PT (*P*<0.05). Compared with the undisturbed soil (NT), RT increased the SR of SOC for 0–5:5–10 cm without statistical significance but significantly decreased the SR of SOC for the other layers (10–20, 20–30, and 30–50 cm) (*P*<0.05). Furthermore, the SRs of SOC for 0–5:5–10, 0–5:10–20, 0–5:20–30, and 0–5:30–50 cm layer followed the order of NT>RT>PT, indicating that the SR of SOC decreased with increased tillage intensity. At the maize harvest, SR of SOC was significant higher under NT than that under PT for 0–5:5–10, 0–5:10–20, 0–5:20–30, and 0–5:30–50 cm. However, there was no difference in the SR of SOC between NT and RT after the maize season. The SR ranged from 1.12 to 3.56, 1.10 to 3.18, and 1.01 to 2.51 for NT, RT, and PT, respectively across the depth ratios. When compared with the wheat harvest, the SR at the maize harvest changed little for the 0–5:5–10 and 10–20 cm depths but decreased for the 0–5:20–30 and 0–5:30–50 cm depths.

**Table 3:** Stratification ratio (SR) of the soil organic carbon (SOC) and the total nitrogen (TN) for the wheat and maize seasons.

		Maize season			Wheat season		
	Soil depth ratio (cm:cm)	NT	RT	PT	NT	RT	PT
SR of SOC	0–5:5–10	1.12Da	1.10Dab	1.01Cb	1.07Dab	1.13Da	1.03Db
	0–5:10–20	1.90Ca	1.70Ca	1.37Cb	2.02Ca	1.67Cb	1.34Cc
	0–5:20–30	2.40Ba	2.36Ba	1.99Bb	2.89Ba	2.44Bb	2.23Bc
	0–5:30–50	3.56Aa	3.18Aa	2.51Ab	3.95Aa	3.41Ab	2.98Ac
SR of TN	0–5:5–10	1.20Da	1.19Da	1.04Db	1.04Ca	1.15Da	1.10Da
	0–5:10–20	1.80Ca	1.72Ca	1.42Cb	1.45BCb	1.72Ca	1.33Cb
	0–5:20–30	2.22Bb	2.85Ba	2.00Bb	2.02Bb	2.53Ba	2.03Bb
	0–5:30–50	2.87Ab	3.12Aa	2.70Ab	3.82Aa	2.91Ab	2.43Ab

NT, no till; RT, rotary tillage; PT, plow tillage. The data are presented as the means (n = 3). The various capital letters indicate significant differences among the soil depths, and the lowercase letters indicate significant differences among the treatments (P<0.05).

The SR of TN also increased significantly with the increase in the soil depth both at the wheat and the maize harvest (Table 3) for all treatments (*P*<0.05). However, the differences between the SR for 0–5:10–20, 0–5:30–50 and 0–5:20–30 cm were not statistically significant under NT. At the wheat harvest, the SR values of TN for 0–5:5–10, 0–5:10–20, and 0–5:20–30 cm were the highest under RT (1.15, 1.72, and 2.53) and for 0–5:30–50 cm under NT (3.82). The SR of TN decreased under PT by 0.05 (with no significant difference), 0.39, and 0.50 for 0–5:5–10, 0–5:10–20, and 0–5:20–30 cm compared with that under RT; and by 1.39 for 0–5:30–50 cm compared with NT (*P*<0.05), respectively. At the maize harvest, the SRs of TN were significant higher under NT and RT for 0–5:5–10 and 0–5:10–20 cm than that under PT, and were significant higher under RT than that under NT and PT for 0–5:20–30 and 0–5:30–50 cm (*P*<0.05). Additionally, the SR was the lowest under PT with values of 0.16 and 0.15, 0.38 and 0.30, 0.22 and 0.85, and 0.17 and 0.41 lower than NT and RT for 0–5:5–10, 0–5:10–20, 0–5:20–30, and 0–5:30–50 cm, respectively.

## Discussion

The results presented herein show that the SOC concentrations were higher under NT for the 0–5 cm depth and decreased more strongly with increases in the soil depth than those in RT and PT for both the wheat and maize seasons, especially for the wheat season (Fig 2). In contrast, the SOC concentration under PT was the highest among the three treatments at the 30–50 cm depth for both wheat and maize seasons. Similar trends have also been reported in several long-term (>10 years) experimental studies [24, 36] as well as in short-term (3 years) experiments [37]. The SR of SOC was higher under NT than that under PT for 0–5: 10–20, 20–30, and 30–50 cm at both seasons. This trend indicates that the conversion of conventional tillage to conservation tillage can expedite the accumulation of SOC in the surface layer. Similar results were reported by Melero et al. [21] and Franzluebbers and Stuedemann [12] because the C input is greater in the surface soil under NT through the surface retention of crop residues. However, RT redistributes crop residues in the top 8–10 cm of soil, whereas, PT incorporates crop residues throughout the plow layer by mechanical operations and increase SOC concentrations in this soil depth.

A stratification ratio of the soil organic C and N pools of 2 is an indicator of improvement in soil quality [2]. In the present study, the SR of SOC for 0–5:10–20, 20–30 and 30–50 cm were all >2 under NT. However, the SR under RT and PT was only >2 below 20 cm at the wheat harvest (Table 3). Furthermore, the SR of SOC and N progressively increases with increases in the soil depth, due to the decrease in the SOC concentrations along the soil profile [24]. Compared with PT, the stratification of SOC with depth is a spontaneous process that is mainly induced by a continuously higher input of C at the soil surface and less in the subsoil under NT treatment [2, 3]. In general, the SR value of SOC and TN (Table 3) increased significantly with increases in the soil depth (*P*<0.05) under all treatments. Among all treatments, the mean SR of SOC under NT was 0.32 and 0.16 higher than that under RT, and 0.59 and 0.53 higher than that under PT in the wheat and maize season, respectively. The effect of the soil sample depth on SR is evident in the present study, but more studies are necessary to determine the appropriate depth of sampling for analyzing the soil properties to compute precisely the SR. Melero et al. [21] observed the relative proportion of variation within the factors contributing to the variations (tillage 54±15%, soil depth increment 25±14%, crop rotation 13±7%, and N fertilizer rate 8±3%) in SR of SOC, TN, etc. Furthermore, the SR of SOC under conservation tillage (NT) was greater than that under conventional tillage especially for the deep depth ratio (PT). The difference among the tillage treatments may be due to the surface application of crop residues under NT. However, SOC is distributed uniformly throughout the tilled layers in RT and PT (0–10 cm under RT and 0–20 cm under PT). Similar results were reported by Sá and Lal [3] and Ferreira et al. [4]. The results presented herein indicate that the SR of TN was higher under NT or RT than that under PT especially at maize season (Table 3). However, the TN concentration was observed no statistical significance at surface soil (i.e., 0–5 cm), and even higher under conventional tillage (PT) than that under conservation tillage (NT) at sub-soil (i.e., 30-50cm) (Fig 3). In addition, conversion to NT increased the SR for 0–20.9:20.9–55.5 and 55.5–102.1 cm of TN compared with that of PT [22]. Moreover, conservation tillage increased the stratification of soil TN compared to conventional tillage [26]. The relatively higher TN concentrations were mainly caused by the undisturbed soil profiles and the application of crop residues at the topsoil under NT.

**Fig 4:**
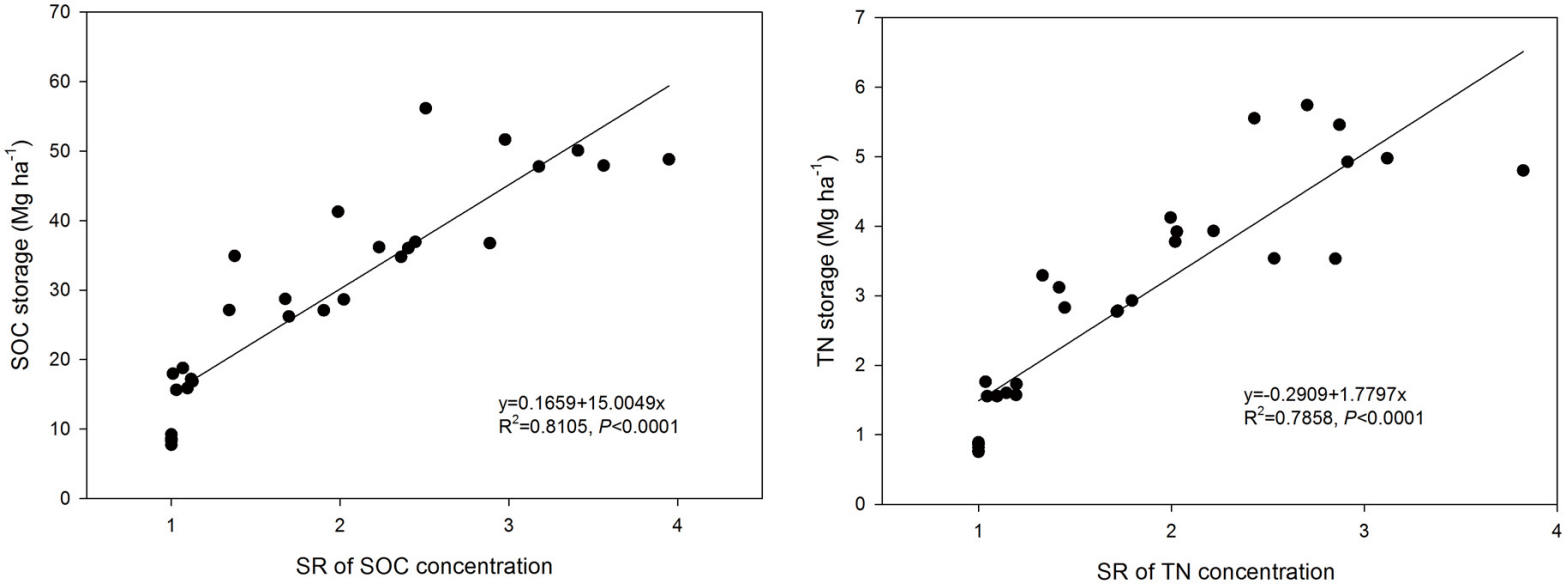
Stratification ratio (SR) related to storage of (A) soil organic carbon and (B) nitrogen.

In addition, the SOC and TN storage were related to the SR (Fig 4). With the increase of SR, the SOC storage (R^2^ = 0.8105, *P*<0.0001) and TN storage (R^2^ = 0.7858, *P*<0.0001) increased significantly. This is mainly due to the trend of SR and the storage of SOC and TN, which were both significantly increased with increased soil depth (Tables 2 and 3). The results indicated that SR could reflect the SOC and TN storage along with the soil profile. As reported by [3], SR could be an indicator of SOC sequestration. The results of SOC storage here illustrated the trend that NT enhanced the surface-soil SOC storage, although without an obvious difference from that under PT in the entire soil profile (e.g., 0–50 cm). Similar results were also observed in previous studies in the NCP [13, 19, 28]. Additionally, the storage of SOC and TN was greater under PT than that that under NT in the deep soil. In addition, SOC storage was higher under PT at the 0–20, 30, and 50 cm soil depth than that under NT or RT at the wheat harvested (Table 2). A global meta-analysis indicated that SOC storage significantly higher under CT than that under NT at 20–40 cm soil depth [15]. The difference in soil profile may be due to an increased C input, decreased soil disturbance and SOC mineralization under NT compared to PT in surface soil [24, 28], and the interactive effect of the plow pan (i.e., altering soil aeration and water transmission) and crop residues retention blew the 20 cm soil depth. In our study, the storage of SOC and TN were calculated by the equivalent soil mass method, which avoided the deviation caused by the variation of ρ_b_ due to dissimilar tillage practices [13, 38] and can reflect the change of the SOC and TN storage more accurately for the various tillage treatments.

Most previous studies have been primarily focused on the depth distribution and relationship of soil C and N but rarely on the factors affecting the SR (e.g., the soil type and structure, soil environment, climate conditions, crops and more), including differences among the crop rotations. Indeed, it is challenging to accurately evaluate the magnitude of the soil properties under the multitude of climatic regions and soil types, thereby making it difficult to identify the effects of crop management on soil ecosystems [2]. In addition, there is a positive relation between the texture and SOC for a wide range of soils, possibly due to the stabilization of organic compounds by clay particles [39]. Furthermore, the SOC stratification within each profile varies with the different types of paddy soils because they relate to the depth distribution of clay and iron oxyhydrates [40]. Thus, further research is needed to understand the mechanisms and factors affecting the dynamics of SOM and SR in the diverse agro-ecosystems of the NCP.

Additionally, the results presented herein illustrated that SR reflected the change of SOC and TN storage responding to tillage practice change. Conversion to NT enhanced the SR of SOC and TN compared to PT, but the SOC and TN concentration and storage were only found higher at top soil and lower at deep soil or the whole soil profile (e.g., 0–50 cm) under NT than that under PT. Moreover, stratification of SOM at surface soil is a key to SOC sequestration and soil quality [2, 3]. The increased SR of SOC and TN may indicate a higher SOM sequestration and soil quality under NT than that under PT in the NCP. Therefore, SR of SOC and TN could be used as an indicator of soil quality and reflected the different changes responded to tillage practices in the NCP. As for soil sampling depth, a previous study shown that SOC will barely change blow 50 cm soil depth in the NCP [13]. Thus, a soil depth of 50 cm may be a better bottom soil layer to calculate SR of SOC. A higher SR values under NT, which indicated a relative abundant SOM at surface soil can enhance soil microbial, enzymatic activities and material exchanges [2, 36, 41]. Surface stratification of SOC, TN can enhance the soil surface functions, while nutrient in subsoil is also important for crop growth. Thus, an efficient nutrient management strategy should include a proper nutrient supplement e.g., a deep fertilization. Therefore, further research on integrated nutrient management under NT is still needed in the NCP.

## Conclusions

A long-term experiment was conducted to assess the changes in SOC and TN and their storage and stratification ratio in relation to tillage systems and to document the effect of tillage on the soil quality in the NCP. The concentrations of SOC and TN decreased with increased soil depth, but an opposite trend was observed in their SR at both the wheat and maize harvests. The SR of SOC was higher under NT and RT than that under PT, especially when compared with the deeper soil.

The SR of SOC under NT and RT were significantly higher than that under PT (*P*<0.05), and the arithmetical mean values of the SR for 0–5:5–10, 10–20, 20–30 and 30–50 cm under NT were higher by 0.32 and 0.16 than those under RT in the wheat and maize seasons, however by 0.59 and 0.53 than those under PT in the wheat and maize seasons, respectively. The SR of TN ranged from 1.04 to 3.82 and 1.20–2.87 under NT, 1.15 to 2.91 and 1.19 to 3.12 under RT and 1.10 to 2.34 and 1.04 to 2.70 under PT in the wheat and maize seasons, respectively. The SOC and TN storage calculated by the equivalent soil mass method was higher under NT for the surface soil (0–5 or 0–10 cm), although it was higher under PT for the deeper soil (0–30 or 0–50 cm). Furthermore, the increases in the SOC and TN were related to the increase in SR along the soil profile. Therefore, SR could be an indicator of SOC and TN storage, reflecting the dynamics of the SOC and TN storage along the soil profile, and the transition to NT increased the SOC and TN in the surface layer, improved the SR of the soil properties through stratification of the SOC and N, and enhanced SOC sequestration in the NCP.
